# Young Men Wanted

**Published:** 1910-08-15

**Authors:** Fred Milton Smith


### YOUNG MEN WANTED.

BY FRED MILTON SMITH, D.D.S.

Places are being vacated daily by men whose desire has been to do a man's work in a manly way and whose thought has been not first a dentist, afterwards a man, but the reverse.

Sometimes a man is so slight and diffident that he is almost lost in the crowd of those who not only believe that "charity begins at home," but that her sweet face must always content herself by his fireside. This man-dentist is not always found in the luxuriously appointed office in one of the commercial centers. If however, he is found there, he may have seated in his chair, recently vacated by the man of wealth and culture, a little child, whose only claim upon his professional skill is, that the child is "one of the least of these." Nor will he be one whit less gentle or sympathetic because his fee is paid in coin not current in commercial circles. If he is asked whether his lucrative practice and social position give him comfort and satisfaction he will probably answer "To be able to meet my just debts without worry makes me happy only in that I am able to give closer attention to the honest day's work that I am seeking to do."

While the places occupied by such men in the cities are sometimes vacated and afford excellent opportunities, let not the young man congratulate himself that if he attains to one of them he has secured through passage in a "Pullman" to Contentment Island. Let him rather remember that to do a man's work in a great city is heroism, and not child's play. Some one has said "of him to whom much is given, shall much be required."

Many, and often overlooked fields are, however, to be found where the early mornings in springtime are filled with the perfume of flowers and the singing of birds, and where the oriole builds her nest just outside the office window. There the dentist whose business it is to do a man's work—and who does it—is judged by what he is and does for his fellow men and the community in which he lives.

Often the occupant of such a place has arrived at the t me when "the grinders cease because they are few, and those that look out of the window be darkened." He is simply awaiting the time "when the silver cord shall be unloosed" and the spirit shall enter into that rest which is reserved for the man who has faithfully finished his day of honest toil. He has spent his life almost entirely in the quiet town. His years of practice have been many and he has not always received the gratitude he deserves. He has taken in hand the poor boy and has as conscientiously cared for him as if he were the son of a wealthy father. If he has rendered any bill it has been a nominal one. He has put forth every effort, using money that he could ill afford in subscribing for journals, and attending society meetings, that he might perfect himself for service to that boy and others. He has rendered a class of service of which nine out of ten men in the larger cities might be proud, and over which many of the more conscientious would blush because of their inability to equal. He has seen the poor boy grow from a poor lad to a wealthy man, and then has been pained to see him seek the services of a prominent city dentist who must be a good man because his fees are large. He worries not so much at the loss of the patient, as because he knows that the dentist to whom his patient has gone can not compare with him in ability. Did this dentist give up, and become discouraged? Not at all. This was only an incident. Very likely in the evening of the day on which he learned of the loss of the patient, he attended a meeting which had for its object the betterment of the conditions in his community. While men of wealth were there, and politicians "of the baser sort," when need called for a man of integrity and intelligence to lead, he was chosen.

When young men in his town wanted to consult with some one who was absolutely unselfish and judicious it was he who was sought out, and from nothing did he derive more pleasure than from the thought that he was useful. Has he been a successful dentist? Well, that depends. If success means to hold all the offices in the dental societies within his reach, no. If it means the accumulation of large sums of money, no. If it means a world wide reputation gained by constantly keeping onesself to the front, no. If, however, it means to live each day as a man should; to lose no opportunity to be helpful to others; to raise and educate a family of children so that they shall be helpful to posterity; to come to three score years and ten and have one hundred men in his own profession meet to do him honor, every one of whom were in hearty accord with the key note sounded by those chosen to speak, namely: "We come together to do honor to an excellent practitioner, and a man whose character after half a century of professional life is without blemish." If this is success, then most emphatically, yes. Young men are wanted for such places—*The Journal.*

